# Design of Technological Parameters for Vibrocompression of Gypsum Concrete

**DOI:** 10.3390/ma18163902

**Published:** 2025-08-20

**Authors:** Leonid Dvorkin, Vadim Zhitkovsky, Yuri Ribakov

**Affiliations:** 1 Department of Building Elements Technology and Materials Science, National University of Water and Environmental Engineering, 33028 Rivne, Ukraine; l.i.dvorkin@nuwm.edu.ua (L.D.); v.v.zhitkovsky@nuwm.edu.ua (V.Z.); 2 Department of Civil Engineering, Ariel University, Ariel 40700, Israel

**Keywords:** gypsum, concrete, vibropressing, design, strength, experiment planning, model

## Abstract

This paper deals with a method for producing gypsum concrete by vibropressing ultra-stiff concrete mixtures with a water–gypsum ratio (W/G) of 0.25–0.35 (stiffness 50–55 s according to Vebe), as well as the method of designing the composition of such concrete. The research was carried out using mathematical experimental design. Experimental and statistical polynomial models of strength and average density dependences on technological factors such as moisture content in the gypsum concrete mixture, aggregate consumption, and vibropressing parameters (dynamic punch pressure during vibration and process duration) were obtained. Models of the aggregate quantity and granulometric composition influence on the gypsum concrete strength at constant compaction parameters and changes in the mixture moisture content were obtained. Based on the obtained models, a method for designing the composition of vibropressed gypsum concrete on dense aggregate was developed. According to the proposed method, the aggregate-to-gypsum ratio (A/G) is first found, taking into account the given strength and quality of the materials. Next, the optimal W/G ratio, which ensures maximum compaction, is calculated and, after that, the residual air volume and the component consumption are obtained. The method allows determining the composition of gypsum concrete on dense aggregate, compacted by vibropressing of superhard mixtures according to a given compressive strength after 1 day of hardening in the range from 15 to 44 MPa. It also allows you to take into account the operating parameters of the molding plant, the aggregate grain composition, and determine the optimal moisture content of the gypsum concrete mixture.

## 1. Introduction

To enhance the performance characteristics of concrete based on gypsum binders, technological solutions that ensure both high compaction and limited water demand are of critical importance. Two effective approaches are generally recognized in this context:

- Forming gypsum products by casting with addition of superplasticizer admixtures [1,2];
- Forming gypsum products from stiff mixes by means of vibrocompression [3,4,5].

In most cases, gypsum-based construction materials are produced from mixtures with a water-to-gypsum (W/G) ratio of 0.5–1.0, typically by casting [6,7,8]. Such processes are characterized by relatively low labor and energy consumption. However, during hardening, only about 18.6% of the hemihydrate mass reacts with water. As a result, the formed products retain high moisture content and must be dried, which leads to increasing fuel and energy consumption as well as extending the overall technological cycle by 6–24 h [7]. Water evaporation leads to pore formation, with pore volume in gypsum products reaching up to 60% and up to 90% of those being capillary pores [8]. Porosity and pore structure have a significant impact on technical properties, reducing strength and increasing hygroscopicity.

The water demand of molding mixtures and the moisture content of gypsum products can be reduced and strength increased through various measures:

- Production and use of gypsum binders with relatively low water demand, such as high-strength gypsum (α-hemihydrate) [8];
- Optimal selection of binder grain size distribution to ensure tight packing and reduced intergranular voids, thereby lowering water demand [8];
- Introduction of admixtures such as plasticizers and superplasticizers [9].

Few studies have focused specifically on stiff gypsum concrete mixes [7,10,11]. However, their substantial technical and economic advantages have spurred increased interest in technologies for forming such stiff mixtures, and several solutions have been proposed. The following methods have been developed for producing gypsum-based materials from low-water-content mixtures:

- Pressing stiff mixtures made of building or high-strength gypsum at W/G = 0.15–0.20; the molding mixture may be prepared by moistening the binder, granulation followed by pressing, or by incorporating porous water-saturated fillers, microcapsules with liquid phase, mineral or organic fibers, etc. [12];
- Forming from plastic and castable mixtures (W/G ≥ 0.5) followed by removal of part of the liquid phase under pressure in a mold, a method first proposed in 1977 [13];
- Pressing of ground gypsum stone followed by hydrothermal treatment, either in an autoclave or directly in the mold [14].

It has been demonstrated [15] that gypsum concrete can be made by pressing stiff mixtures at W/G = 0.15–0.20. Low water content may also be achieved by pressing plastic mixtures and removing part of the liquid phase under pressure [15]. Pressed material was first obtained from non-fired gypsum binder, and the effect of pressing pressure was studied. It was demonstrated that increasing pressure up to 15 MPa doubles the strength of artificial gypsum stone, while further pressure increase may even reduce strength [16].

The use of semi-dry pressing for gypsum product manufacturing significantly simplifies the technology and reduces energy consumption by eliminating the drying stage. Vibrocompression is especially suitable for high-speed, mass production of small-sized products with specific geometrical parameters and high physical and mechanical properties, ensuring long service life under demanding operating conditions [17]. Gypsum concrete products differ from similar cement-concrete products in being more environmentally friendly, mainly due to significantly lower carbon dioxide emissions. Their low water resistance allows the use of such material in dry conditions. Therefore, the most rational direction of their application in practice is using them for walls placed inside rooms [16].

Many researchers have studied stiff concrete mixes and vibrocompression-based technologies. The development of theory and practice in the field of concrete compaction by vibrocompression has contributed to the wide adoption of this method. A particular feature of vibroforming stiff mixes (with stiffness index over 10 s) is insufficient thixotropic liquefaction [18,19,20]. To enable this effect, the solvated particles of the binder must come into close contact via their water shells. This is achieved through the application of compaction pressure [21].

Key structural features of vibration presses include separate or combined vibration and pressure mechanisms, mix feeding and leveling systems, and non-removable molds allowing immediate demolding and transport of the formed products.

Compacting pressure in different types of vibration presses can be provided either by the punch weight or via pneumatic or hydraulic systems. These presses typically apply a pressure of 0.05 to 0.1 MPa, vibration amplitudes of 0.5–1 mm, and frequencies around 50 Hz, with compaction duration ranging from 5 to 30 s. These forming parameters enable efficient compaction of high-stiffness concrete mixes (Vebe time 50–100 s or more) [22].

Vibration presses are used for both small products (wall blocks, tiles, and paving slabs) and larger items such as rings, pipes, beams, and girders. Thanks to short cycle durations and immediate demolding, this compaction method is highly productive and allows for maximum automation of the production process. The effectiveness of vibrocompression depends on various technological parameters, including the properties and granulometry of aggregates, water–binder ratio, binder content, types and dosages of admixtures, vibration intensity, and compaction pressure [23].

In practice mixture design for vibrocompressed concrete is usually carried out empirically, requiring significant laboratory resources and time. A combined computational and experimental method has been proposed, based on I.M. Akhverdov’s method for designing heavy concrete compositions, taking into account their structural and technological features [24]. This method allows the composition of concrete subjected to vibrocompression to be determined, taking into account the vibration parameters and their effect on the cement stone porosity, and also considers the effect of aggregates on the concrete mix water demand. However, this method does not take into account the peculiarities that arise when using super-stiff (semi-dry) mixtures and considers the behavior of gypsum as a binder in such mixtures.

Thus, the recommendations available in the literature do not allow optimal solutions to be chosen for the compaction degree of gypsum concretes obtained by vibropressing superhard mixtures in industrial conditions. The purpose of the present study is to obtain quantitative strength dependences that take into account the main technological factors and allow design of the necessary compaction modes, compositions of vibropressed gypsum concretes, and also to ensure the specified strength with minimal gypsum consumption. The implementation of this goal determines the novelty of this study.

With this aim a series of experiments was performed according to mathematical plans in order to obtain experimental and statistical models of the influence of various technological factors on the gypsum concrete properties. The hypothesis of this study is that the actual mutual influence of technological factors on the gypsum concrete strength can be determined by experimental and statistical models. A calculation method that allows you to determine the composition of gypsum concrete that is compacted by vibropressing, allowing consideration of the compaction parameters, the aggregate grain composition features and the optimal mixture humidity, which ensures its best molding properties were developed. The efficiency of the proposed method is demonstrated by design of gypsum concrete of different strength classes using various types of gypsum binder.

## 2. Materials

The raw materials used in the research were gypsum binder and quartz sand. The main technical characteristics of the gypsum binder are presented in Table 1.

Two types of quartz sand were used in the present research: sand 1 with a fineness modulus M*_f_* = 2.0 and sand 2 with M*_f_* = 3.0. Sand characteristics are given in Table 2.

Citric acid in the amount of 0.05% of the gypsum mass was used as a setting retarder.

## 3. Methods

The properties of the raw materials and gypsum concrete based on them were determined by standard methods [25,26].

**Figure 1:**
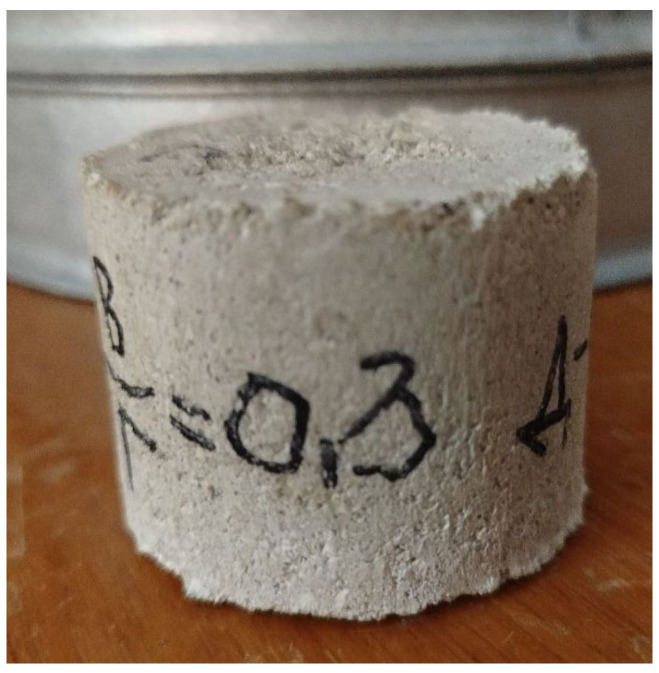
Cylindrical gypsum concrete specimen.

**Figure 2:**
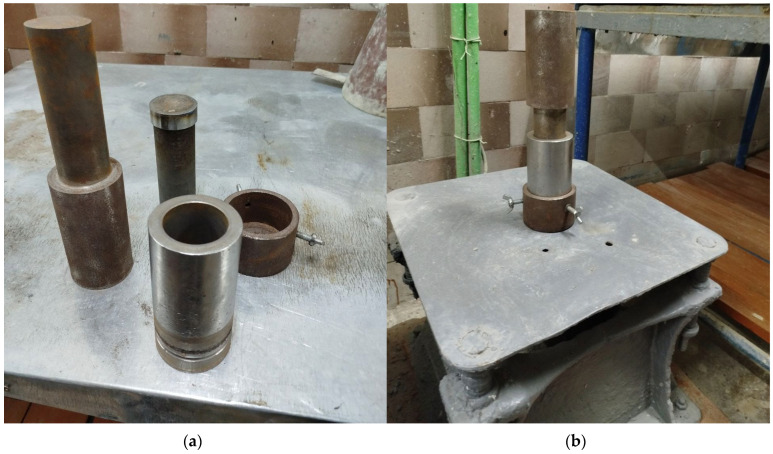
Production of gypsum concrete specimens by vibropressing: (**a**)—mold details; (**b**)—assembled mold on the vibration platform.

Cylindrical specimens with *d* = *h* = 50 mm (Figure 1) were formed by vibrocompaction on a laboratory vibrating platform in a special press mold using appropriate loads (Figure 2). The following compaction parameters typical for most industrial vibrocompactors were selected: frequency—50 Hz and amplitude—0.5 mm. Vibrocompaction duration varied from 5 to 25 s, and the load pressure ranged from 0.012 to 0.112 MPa [27].

### 3.1. Experimental Design

**Figure A1:**
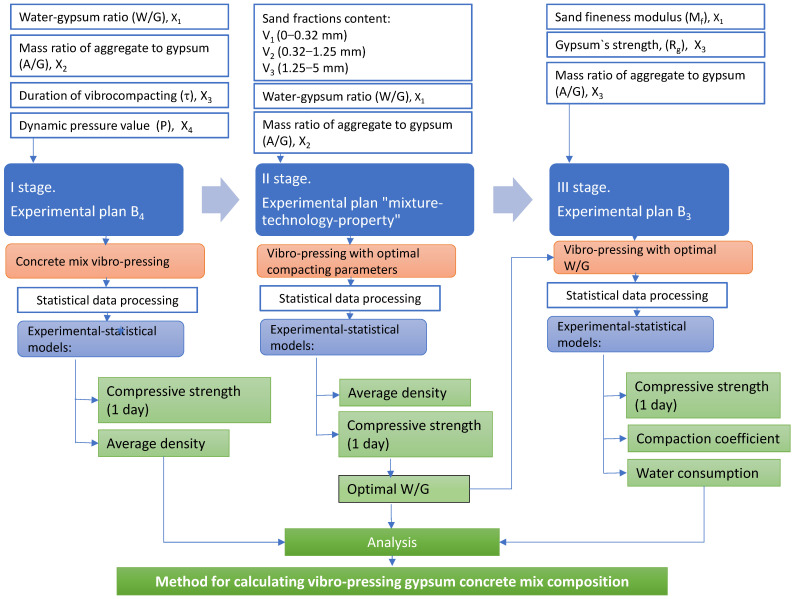
Flowchart of the conducted research.

The studies of gypsum concrete produced by vibrocompaction of a semi-dry concrete mixture were conducted in three stages. For effective implementation of the experiments, formalization of experimental data, technological analysis, and optimization decisions, the method of mathematical–statistical modeling [28,29] was used. The flow chart of the research is given in Appendix A (Figure A1). The experiments were carried out according to the experimental plans. Three samples were tested at each point of the experiment. As a result of statistical processing of the obtained data, experimental–statistical models of the initial parameters were obtained in the form of polynomial regression equations. Each experimental plan contained additional replicate points to verify the adequacy of the resulting equations. The statistical analysis included the following components: a full ANOVA table, a lack-of-fit test, diagnostic graphs (residual vs. fitted, Q-Q, and Cook’s distance), as well as univariate dependences of the output parameters. The resulting statistical characteristics are presented in Appendix B, Appendix C, Appendix D, Appendix E, Appendix F, Appendix G and Appendix H. Statistical analysis of the results and construction of graphical dependencies were carried out using the “Statistica 14.0” software package [30].

### 3.2. The Influence of Mix Moisture, Aggregate Consumption, and Vibrocompaction Parameters

At the first stage of this study, to determine the influence of vibrocompaction parameters on the formation of vibropressed gypsum fine-grained concretes, experiments were carried out according to the B_4_ plan [26] under the planning conditions provided in Table 3. As the output parameters the average concrete density (ρ_0_, kg/m^3^) and compressive strength at the age of 1 day (f_c_^1d^, MPa) were taken. The gypsum concrete compositions used for specimens’ production and experimental results are presented in Table 4.

As a result of statistical processing of the experimental results (Table 4), regression equations for the output parameters were obtained:

ρ_0_ = 1843 + 158 X_1_ +137 X_2_ + 23 X_3_ + 83 X_4_ − 51 X_1_^2^ − 40 X_2_^2^ − 19 X_3_^2^ − 9 X_4_^2^ − 48 X_1_X_2_ − 31 X_1_X_3_ − 30 X_1_X_4_ + 9 X_2_X_3_ − 15 X_2_X_4_ − 9 X_3_X_4_ (1)

f_c_^1*d*^ = 12.25 + 2.19 X_1_ − 2.49 X_2_ + 0.96 X_3_ + 1.64 X_4_ − 4.07 X_1_^2^ + 1.05 X_2_^2^ − 1.76 X_3_^2^ − 2.42 X_4_^2^ − 0.83 X_1_X_2_ + 0.59 X_1_X_3_ + 0.66 X_1_X_4_ − 0.16 X_2_X_3_ − 0.08 X_2_X_4_ + 0.08 X_3_X_4_ (2)

The statistical indicators of the experiment and the obtained Equations (1) and (2) are given in Appendix B and Appendix C.

### 3.3. Granulometric Composition at Constant Compaction Parameters and Changes in Mix Moisture

The combined effect of the aggregate grain composition and its quantity on the gypsum concrete strength was studied under constant compaction parameters and varying mixture moisture. For this purpose, experiments were performed following the “mixture-technology-property” plan [26]. The planning matrix and experimental results are presented in Table 5.

The influence of sand fraction content on the strength of vibropressed gypsum concrete was studied:

V_1_—0–0.32 mm (5–35%); V_2_—0.32–1.25 mm (40–70%); V_3_—1.25–5 mm (25–55%), at X_1_—mass ratio of aggregate to gypsum, (A/G)—1–3; X_2_—water–gypsum ratio (W/G)—0.15–0.35.

During the experiments, the specified molding parameters were maintained: frequency—50 Hz, amplitude—0.5 mm, vibrocompaction duration—15 s, and load pressure—0.06 MPa [22].

After statistical processing of the experimental results, a mathematical model of the strength of vibropressed gypsum concrete was obtained:

(3) $$\begin{array}{c} f_{c}^{1d}=10.68V_{1}+12.76V_{2}+10.11V_{3}+11.69V_{1}V_{2}+46.95V_{1}V_{3}+30.03V_{2}V_{3}-\\ 20.7V_{1}X_{1}+6.67V_{1}X_{2}+2.87V_{2}X_{2}-4.74V_{1}X_{1}+5.48V_{3}X_{2}+\\ 2.77X_{1}X_{2}+0.71X_{1}^{2}-8.09X_{2}^{2} \end{array}$$

(4) $$\begin{array}{c} \rho_{o}=2297V_{1}+2154V_{2}+2271V_{3}-256V_{1}V_{2}+241V_{1}V_{3}+72V_{2}V_{3}+\\ +93V_{1}X_{1}+129V_{1}X_{2}+90V_{2}X_{1}+128V_{2}X_{2}-11V_{3}X_{1}+198V_{3}X_{2}+\\ +52X_{1}X_{2}-73X_{1}^{2}-72X_{2}^{2}\; \end{array}$$

The statistical indicators of the experiment and the obtained Equations (3) and (4) are given in Appendix D and Appendix E.

### 3.4. The Influence of Mix Composition Parameters at Optimal Moisture

To establish the relationship between strength and the main parameters of vibropressed gypsum concrete (VPGC) at the optimal water-to-gypsum ratio (W/G), an experiment was conducted according to B_3_ plan [26]. The experimental design conditions are presented in Table 6.

The experiments employed compaction parameters typical of most industrial vibropresses: frequency—50 Hz, amplitude—0.5 mm, pressure—0.06 MPa, and compaction duration—15 s. The output parameters included the following characteristics: water consumption (W, l/m^3^), compaction coefficient (K_c_), and compressive strength at 1 day of age (f_c_^1d^, MPa) (Table 7).

The optimal W/G was determined based on the best formability of the mix. This was visually monitored by the appearance of the liquid phase on the sample surface. The test results were processed using mathematical statistics, and mathematical models were obtained:

f_c_^1*d*^ = 26.1 – 1.5X_1_ + 7.5X_2_ – 7.5X_3_ – 1.7X_1_^2^ + 2.5X_2_^2^ + 2.7X_3_^2^ – 0.05X_1_X_2_ – 0.2X_1_X_3_ – 2X_2_X_3_ (5)

K_c_ = 0.92 – 0.01X_1_ + 0.031X_2_ – 0.009X_3_ – 0.003X_1_^2^ – 0.001X_2_^2^ – 0.011X_3_^2^ – 0.001X_1_X_2_ – – 0.012X_1_X_3_ + 0.01X_2_X_3_ (6)

W = 90.3 + 4.9X_1_ – 16.5X_2_ – 18.6X_3_ + 1.0X_1_^2^ – 1.9X_2_^2^ + 4.3X_3_^2^ + 0.4X_1_X_2_ – 1.2X_1_X_3_ + 6/7X_2_X_3_ (7)

The statistical indicators of the experiment and the obtained Equations (5)–(7) are given in Appendix F and Appendix H.

## 4. Results Analysis

### 4.1. The Influence of Mix Moisture, Aggregate Consumption, and Vibrocompaction Parameters

Following the obtained data (Table 4, Equation (1)), the compressive strength of vibropressed gypsum concrete varies widely (from 5.3 to 17 MPa), which can be explained by various reasons. Factor X_1_ (W/G) has an almost linear influence on strength when W/G ≥ 0.25; factor X_2_ (A/G) has a sharply negative linear effect; factors X_3_ and X_4_, which characterize vibrocompaction duration and pressure, positively affect the strength.

**Figure 3:**
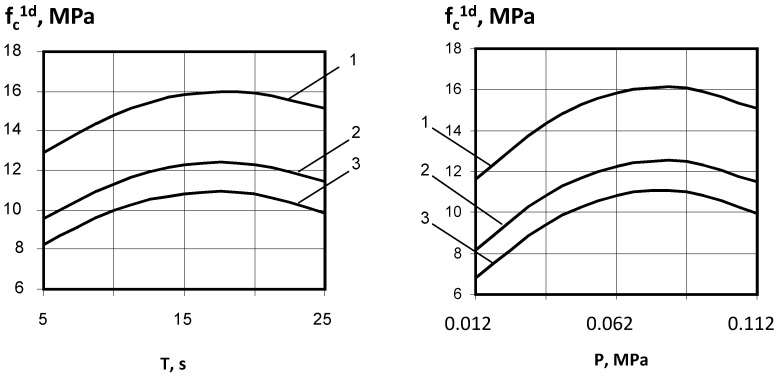
Dependence of the vibropressed gypsum concrete strength on compaction parameters: 1—A/G = 0; 2—A/G = 1; 3—A/G = 2.

Factor X_4_ has a particularly noticeable effect, with a smaller effect from X_3_ (Figure 3). Negative values of the quadratic effects of X_3_ and X_4_ indicate the existence of an optimum range of compaction parameters within the variation range, closer to the upper level (T = 15…20 s, P = 0.06 … 0.09 MPa). Thus, for vibropressed gypsum concrete, an optimal range of loading pressure and vibrocompaction duration was established, which ensures maximum density and strength of concrete. The obtained data are confirmed by similar results obtained for cement-based vibropressed concrete [22].

The average density values of vibropressed gypsum concrete range from 1400 to 2000 kg/m^3^ (Table 4, Equation (2)). The greatest increase in ρ_0_ is caused by an increased share of aggregate; mixtures with less binder are more prone to compaction due to the vibration-induced movement of coarse particles and their compact arrangement.

**Figure 4:**
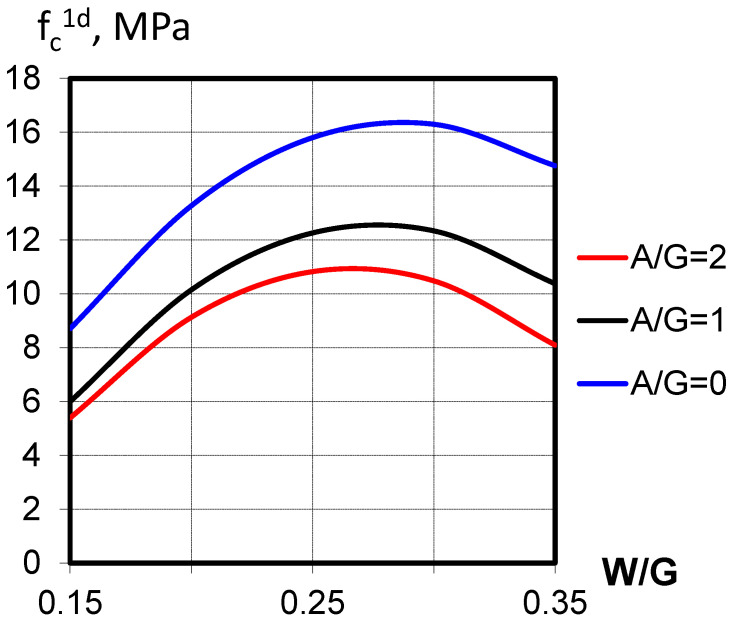
Influence of water–gypsum ratio (W/G) on the vibropressed gypsum concrete strength (f*_c_*^1*d*^).

The optimal W/G under the given conditions ensures the necessary compaction, sufficient water for hydration, and minimal porosity. When W/G is below optimum, the gypsum concrete mixture becomes too stiff and cannot be properly pressed under the given vibrocompaction parameters (Figure 4). At the same time, the low amount of water causes a rapid transition of gypsum to dihydrate, possibly preventing full hydration. Increasing the water content to optimum facilitates more uniform mixing and wetting, reduces viscosity to the necessary level, and prolongs the setting time, allowing the formation process to be completed during the induction period. Further increase in water content in the mixture leads to increased porosity of the gypsum matrix and technological defects (adhesion of products to the punch and mold walls and deformation).

### 4.2. Granulometric Composition at Constant Compaction Parameters and Changes in Mix Moisture

Given that, along with the water–gypsum ratio, the content of aggregate significantly affects the vibropressed gypsum concrete strength, it was deemed appropriate to determine the influence of the aggregate’s grain composition depending on W/G and the aggregate-to-gypsum ratio (A/G).

**Figure 5:**
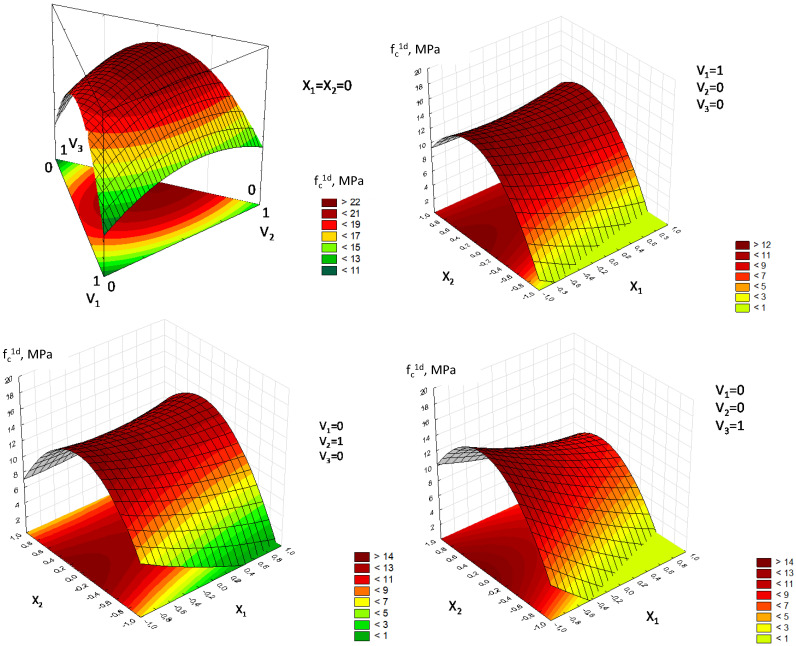
Response surfaces of compressive strength of vibrocompacted gypsum concrete, obtained using the experimental–statistical model (3), depending on the variation of factors: V_1_—0–0.32 mm; V_2_—0.32–1.25 mm; V_3_—1.25–5 mm; X_1_—mass ratio of aggregate to gypsum, (A/G); X_2_—water–gypsum ratio (W/G).

Analysis of Equation (3) (Figure 5) shows that the strength of vibropressed gypsum concrete significantly depends on the aggregate grain composition. There is an optimal combination of fractions that ensures the maximum strength of gypsum concrete, determined by the minimum voidity of the aggregate. The influence of factor X_2_ (W/G) is characterized by a significant negative quadratic effect, indicating its extreme influence on compressive strength.

As can be seen from the response surfaces constructed using Equation (3), the optimal W/G value ((W/G)*_opt_*) varies depending on the aggregate content and its grain composition (Figure 5). To develop a model of the optimal (W/G)_opt_, Equation (3) was differentiated with respect to the X_2_ variable (W/G):

(8) $$\frac{\partial R_{c}^{1d}}{\partial X_{2}}=6.67V_{1}+2.87V_{2}+5.48V_{3}+2.77X_{1}-16.18X_{2}$$

To find the value of X_2_ at maximum f*_c_*^1*d*^, a condition was written:

(9) $$\frac{\partial R_{c}^{1d}}{\partial X_{2}}=0$$

(10) $${\left(X_{2}\right)}_{R_{c}^{\max}}=0.41V_{1}+0.177V_{2}+0.34V_{3}+0.17X_{1}$$

The analysis of the resulting equation confirms the linear influence of the grain composition factors (V_1_–V_3_) and the aggregate-to-gypsum ratio (X_1_) on the W/G value (X_2_) that provides the maximum compressive strength. Among the grain composition factors, V_1_ and V_2_ (fine and medium fraction content) have the greatest influence on the optimal W/G value (X_2_) (Figure 3).

By converting Equation (10) to the natural form, a mathematical model of the W/G optimal from the perspective of maximum strength was obtained:

(11) WGopt=0.041V1+0.0177V2+0.034V3+0,017AG+0.216

**Figure 6:**
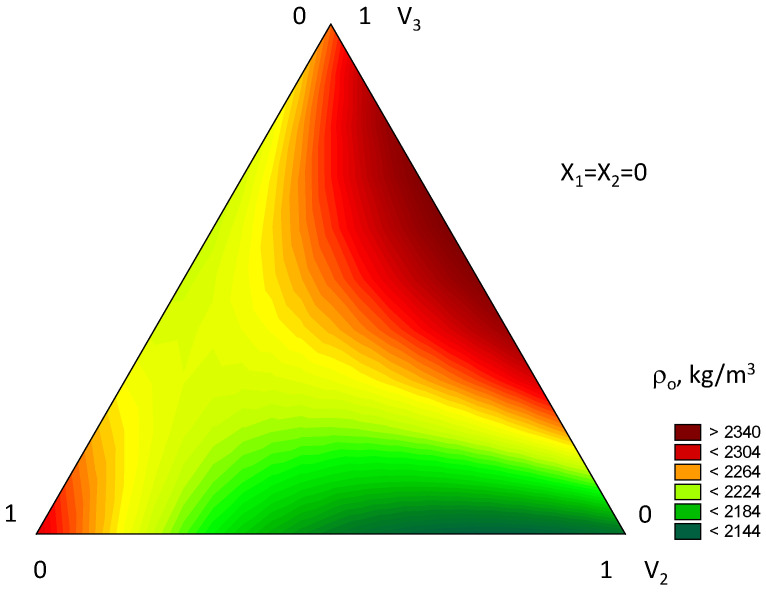
Isoparametric diagram of the change in average density (ρ_o_, kg/m^3^) of vibropressed gypsum concrete depending on the aggregate gradation: V_1_—0–0.32 mm; V_2_—0.32–1.25 mm; V_3_—1.25–5 mm; X_1_—mass ratio of aggregate to gypsum, (A/G); X_2_—water–gypsum ratio (W/G).

The analysis of the mathematical model of the average density of gypsum concrete (Equation (4), Figure 6) reveals both individual and combined effects of the varied factors. Among the grain composition factors (V_1_–V_3_), the most significant is v1 (fine fraction 0–0.315 mm), followed closely by V_3_ (coarse fraction 1.25–5 mm). Factor V_2_ (medium fraction 0.315–1.25 mm) causes a decrease in the output parameter by 120–140 kg/m^3^.

Increasing the water-to-gypsum ratio of the mix up to certain values leads to increased density of vibropressed gypsum concrete due to reduced mixture viscosity and improved compactability. The minimally effective W/G from the standpoint of achieving maximum density ranges from 0.28 to 0.33.

### 4.3. The Influence of Mix Composition Parameters at Optimal Moisture

**Figure 7:**
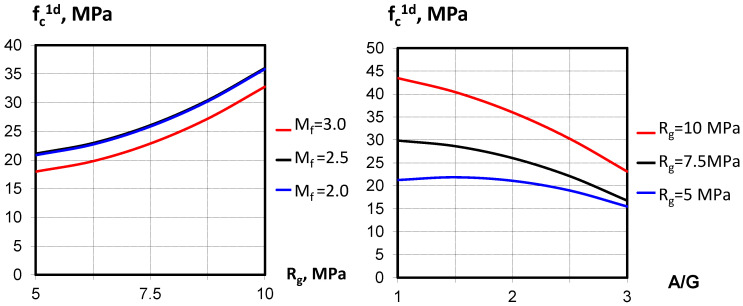
Dependence of vibropressed gypsum concrete strength (f_c_) on the investigated factors at optimal water-to-gypsum ratio (W/G)ₒₚₜ.

The strength of VPGC at the optimal W/G varied from 10 to 45 MPa (Table 5, Equation (7), Figure 7). The most significant influence was exerted by the binder strength and the aggregate content. The considerable interaction coefficient between these factors indicates that, with higher aggregate content, the influence of binder strength decreases. An increase in binder strength leads to a reduction in water demand (Equation (8)), which, in turn, increases K_c_ (Equation (9)), allowing compressive strengths of 35–45 MPa.

### 4.4. Method for Calculating Vibropressed Gypsum Concrete Mix Composition

The obtained relationships between strength and water demand enable the development of a method for calculating VPGC mix composition:

1. Based on the strength model (Equation (5)), the ratio of aggregate to gypsum (A/G) is determined according to the given strength. This takes into account the gypsum’s strength of the fineness modulus of sand. Equation (5) in its natural form is as follows:

$$f_{c}^{1d}\;=\;2.7{\left(A/G\right)}^{2}-0.4\left(A/G\right)M_{f}\;-0.8\left(A/G\right)\;R_{g}-11.3\left(A/G\right)-6.8\;M_{f}^{2}-0.04\;M_{f}R_{g}\;+32.1M_{f}+0.4R_{g}^{2}-1.3R_{g}+2.15$$

2. From the model (Equation (11)), taking into account the A/G and the particle size distribution, the optimal W/G ratio ((W/G)ₒₚₜ) is as follows:

WGopt=0.041V1+0.0177V2+0.034V3+0,017AG+0.216

Using Equation (9), K*_c_* is determined for the given fineness modulus of sand, binder strength, and A/G ratio, and the predicted entrapped air content in the concrete (V*_e_*_.*a*._, l) is calculated.

Equation (9) in its natural form is as follows:

K_c_ = −0.011⋅(A/G)^2^ − 0.024⋅(A/G)⋅M_f_ + 0.004⋅(A/G)⋅R_g_ + 0.065⋅(A/G) − 0.012⋅M_f_^2^ −

0008⋅M_f_⋅R_g_ + 0.094⋅M_f_ − 0.00016⋅R_g_^2^ + 0.0088⋅R_g_ + 0.692

Equation Ve.a.:

$$V_{e.a.}=\left(1-K_{c}\right)\cdot 100$$

3. The gypsum content is then calculated.

$$G=\frac{1000-V_{e.a.}}{\frac{1}{\rho_{g}}+\frac{W}{G}+\frac{\left(A/G\right)}{\rho_{a}}}$$

4. The water consumption is determined:

W=G⋅WG;

5. The aggregate consumption is determined:

A=G⋅AG

The results of the VPGC mix design calculations are presented in Table 8.

The obtained concrete mix composition is refined by performing laboratory trial batches.

## 5. Conclusions

1. The influence of the humidity of the superhard gypsum concrete mixture (50–55 s according to Webe), aggregate content, dynamic pressure of the punch, and the compaction duration on the average density and strength of gypsum concrete was investigated. Experimental–statistical polynomial models of the dependence of these properties on technological factors were constructed.
2. It was found that, at a vibration frequency of 50 Hz, maximum density and compressive strength in the range from 11 to 16 MPa are achieved at a punch pressure of 0.06–0.09 MPa, a compaction duration of 15–20 s, and an optimal water–gypsum ratio (W/G) of 0.26–0.28, which ensures effective compaction, sufficient hydration, and minimal porosity.
3. It has been shown that the fractional composition and content of the aggregate, together with the moisture content of the mixture, significantly affect the density and strength. The optimal combination of fractions is determined by the minimum void content of the aggregate.
4. The obtained equation of the optimal W/G ratio provides maximum compressive strength up to 23 MPa, taking into account the aggregate content and grain composition.
5. The developed models describe the compressive strength in the range from 15 to 44 MPa, the compaction coefficient, and water consumption at optimal mixture moisture content, taking into account the binder strength, the content, and the size of the aggregate.
6. Based on a set of models, a method for designing the composition of vibropressed gypsum concrete from ultra-hard mixtures with dense aggregate is proposed, which allows optimal compositions to be determined for classes from C8/10 to C20/25.
7. In the future, it is planned to expand the range of application of the developed composition design method by adding the possibility of using composite waterproof gypsum binders, as well as lightweight aggregates.

## Figures and Tables

**Table 1:** Technical indicators of gypsum binder [25].

Type of Gypsum Binder	Properties of Gypsum Binder					
	Normal Consistency, %	Setting Time, Min.		Fineness of Grinding (Residue on Sieve 0.2 mm)	Strength, MPa After 2 h.	
		Initial	Final		Compression	Bending
G 5	62	5	10	2.1	5.3	2.7
G 10	55	4	12	1.3	10.8	4.7

**Table 2:** Properties of the sand samples.

Sand Type	Partial Residues, % on Sieves with Opening Sizes, mm						Grain Content <0.16, %	Dust Particle Content, %	Fineness Modulus (M_f_)
	5	2.5	1.25	0.63	0.32	0.16			
Sand 1	0.4	1.8	8.2	13.9	44.0	29.7	2.0	1.2	2.0
Sand 2	1.6	9.6	25.7	33.8	18.6	12.1	1.1	0.2	3.0

**Table 3:** Experiment planning conditions B_4_.

No.	Factors		Variation Levels			Variation Interval
	Natural Type	Coded Type	−1	0	+1	
1	Water–gypsum ratio (W/G)	X_1_	0.15	0.25	0.35	0.10
2	Mass ratio of aggregate to gypsum, (A/G)	X_2_	0	1	2	1
3	Duration of vibrocompacting, (τ, s)	X_3_	5	15	25	10
4	Dynamic pressure value (P, MPa)	X_4_	0.012	0.062	0.112	0.05

**Table 4:** Planning matrix B_4_ and experimental results.

No.	Coded Values of Factors				Composition of Concrete Mix, kg/m^3^			Values of Output Parameters	
	X_1_	X_2_	X_3_	X_4_	Gypsum	Aggregate (Sand)	Water	f_c_^1d^, MPa	ρ_o_, kg/m^3^
1	+1	+1	+1	+1	678	1356	237	7.82	1938
2	+1	+1	+1	−1	678	1356	237	3.85	1910
3	+1	+1	−1	+1	678	1356	237	5.08	1949
4	+1	+1	−1	−1	678	1356	237	1.28	1825
5	+1	−1	+1	+1	1388	0	486	14.17	1708
6	+1	−1	+1	−1	1388	0	486	9.88	1645
7	+1	−1	−1	+1	1388	0	486	10.71	1836
8	+1	−1	−1	−1	1388	0	486	6.33	1714
9	−1	+1	+1	+1	784	1569	118	2.63	1772
10	−1	+1	+1	−1	784	1569	118	1.04	1640
11	−1	+1	−1	+1	784	1569	118	1.92	1730
12	−1	+1	−1	−1	784	1569	118	0.82	1559
13	−1	−1	+1	+1	1922	0	288	5.43	1504
14	−1	−1	+1	−1	1922	0	288	3.51	1225
15	−1	−1	−1	+1	1922	0	288	4.12	1356
16	−1	−1	−1	−1	1922	0	288	2.98	1128
17	+1	0	0	0	911	911	319	9.41	1901
18	−1	0	0	0	1114	1114	167	6.97	1687
19	0	+1	0	0	727	1454	182	7.41	1928
20	0	−1	0	0	1612	0	403	19.23	1683
21	0	0	+1	0	1002	1002	251	11.56	1906
22	0	0	−1	0	1002	1002	251	9.45	1746
23	0	0	0	+1	1002	1002	251	13.45	1915
24	0	0	0	−1	1002	1002	251	6.23	1586
25	0	0	0	0	1002	1002	251	12.25	1830
26	0	0	0	0	1002	1002	251	12.10	1848
27	0	0	0	0	1002	1002	251	12.40	1854

**Table 5:** Planning matrix “mixture-technology-property” and experimental results.

No.	Coded factors Value					Natural Factors Value					Compressive Strength, MPa	Average Density, kg/m^3^
	V_1_	V_2_	V_3_	X_1_	X_2_	Sand Fractions Content, %			A/G	W/G		
						V_1_ (0–0.32 mm)	V_2_ (0.32–1.25 mm)	V_3_ (1.25–5 mm)				
1	1	0	0	−1	−1	35	40	25	1	0.15	1.8	1973
2	1	0	0	+1	+1	35	40	25	3	0.35	10.5	2433
3	0	1	0	−1	−1	5	70	25	1	0.15	5.8	2068
4	0	1	0	+1	−1	5	70	25	3	0.15	1.6	2141
5	0	1	0	+1	+1	5	70	25	3	0.35	8.5	2497
6	0	1	0	−1	+1	5	70	25	1	0.35	10.7	2218
7	0	0	1	−1	−1	5	40	55	1	0.15	5.8	2022
8	0	0	1	+1	0	5	40	55	3	0.25	5.7	2191
9	0	0	1	−1	+1	5	40	55	1	0.35	9.0	2239
10	0	0.5	0.5	0	0	20	55	25	2	0,15	3.0	2071
11	0.8	0.2	0	−1	+1	29	46	25	1	0.35	10.4	2121
12	0.3	0	0.7	+1	+1	14	40	46	3	0.35	19.3	2414
13	0.5	0	0.5	+1	−1	20	40	40	3	0.15	1.1	2047
14	0.6	0	0.4	0	0	23	40	37	2	0.25	21.6	2341
15	0	0.4	0.6	0	+1	5	52	43	2	0.15	5.9	2116
16	0	0.5	0.5	−1	0	5	55	40	1	0.25	23.9	2220
17	0	0.5	0.5	−1	0	5	55	40	1	0.25	24.3	2253
18	0	0.5	0.5	−1	0	5	55	40	1	0.25	23.4	2240
19	0	0.5	0.5	−1	0	5	55	40	1	0.25	22.9	2206

**Table 6:** Experiment planning conditions at the optimal water-to-gypsum ratio.

No.	Factors		Variation Levels			Variation Interval
	Natural Type	Coded Type	−1	0	+1	
1	Sand fineness modulus, (M_f_)	X_1_	2.0	2.5	3.0	0.5
2	Gypsum’s strength, (R_g_, MPa)	X_2_	5	7.5	10	2.5
3	Mass ratio of aggregate to gypsum, (A/G)	X_3_	1	2	3	1

**Table 7:** Planning matrix and experimental results at the optimal water-to-gypsum ratio.

No.	Coded Factors Value			Natural Factors Value			Values of Output Parameters			
	X_1_	X_2_	X_3_	M_f_	R_g_, MPa	A/G	W/G	W, l/m^3^	f_c_^1d^, MPa	K_c_
1	−1	+1	+1	2.0	10	3	0.23	69.6	18.8	0.90
2	−1	+1	−1	2.0	10	1	0.16	95.1	39.2	0.96
3	−1	−1	+1	2.0	5	3	0.29	88.5	12.3	0.86
4	−1	−1	−1	2.0	5	1	0.23	142.4	19.6	0.89
5	+1	+1	+1	3.0	10	3	0.20	60.1	21.9	0.94
6	+1	+1	−1	3.0	10	1	0.14	82.5	42.8	0.96
7	+1	−1	+1	3.0	5	3	0.27	82.2	16.7	0.89
8	+1	−1	−1	3.0	5	1	0.22	129.8	21.6	0.88
9	−1	0	0	2.0	7.5	2	0.24	94.9	23.6	0.91
10	+1	0	0	3.0	7.5	2	0.22	86.5	25.4	0.94
11	0	+1	0	2.5	10	2	0.18	73.1	39.8	0.96
12	0	−1	0	2.5	5	2	0.25	102.5	17.7	0.89
13	0	0	+1	2.5	7.5	3	0.25	75.9	17.5	0.91
14	0	0	−1	2.5	7.5	1	0.19	112.1	29.5	0.92
15	0	0	0	2.5	7.5	2	0.23	90.7	25.7	0.92
16	0	0	0	2.5	7.5	2	0.23	91.0	26.1	0.91
17	0	0	0	2.5	7.5	2	0.23	91.0	25.4	0.93

**Table 8:** Results of the mix design calculation of vibrocompressed gypsum concrete.

Concrete Class	A	A/G	(W/G)_opt_	K_c_	V_e.a._, l	G, kg/m^3^	S, kg/m^3^	W, l/m^3^
C8/10	G5	2.98	0.297	0.88	12.3	551	1643	163
	G7	3.21	0.257	0.91	8.5	527	1690	136
	G10	3.65	0.234	0.95	5.0	484	1766	113
C12/15	G5	2.24	0.284	0.89	10.8	660	1476	187
	G7	2.69	0.249	0.92	7.6	592	1591	147
	G10	3.27	0.227	0.96	4.3	522	1708	119
C16/20	G5	-	-	-	-	-	-	-
	G7	1.99	0.237	0.93	7.3	709	1409	168
	G10	2.84	0.220	0.96	3.9	573	1629	126
C20/25	G5	-	-	-	-	-	-	-
	G7	-	-	-	-	-	-	-
	G10	2.32	0.211	0.96	3.9	650	1510	137

**Figure A2:**
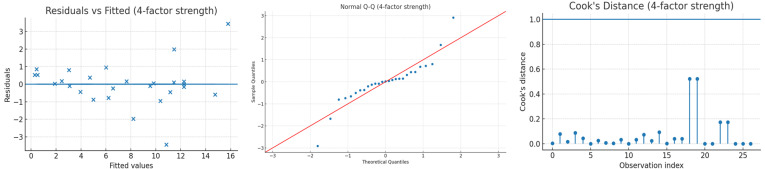
Diagnostic plots for the model (Equation (1)).

**Figure A3:**
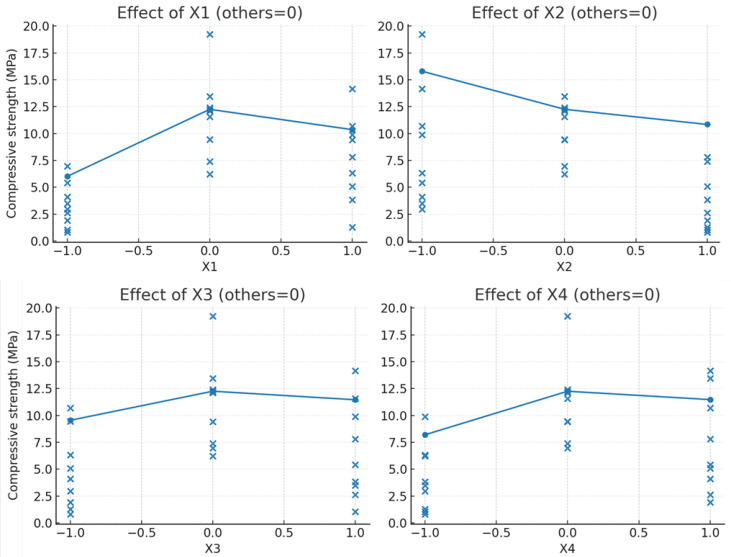
Univariate dependencies to the model (Equation (1)).

**Figure A4:**
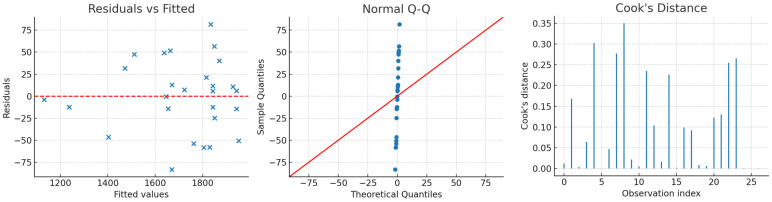
Diagnostic plots for the model (Equation (2)).

**Figure A5:**
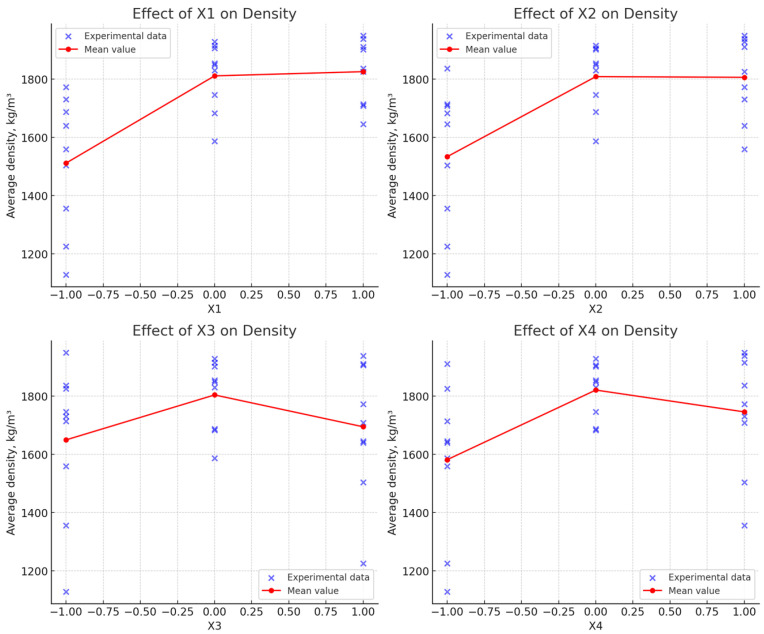
Univariate dependencies to the model (Equation 2).

**Figure A6:**
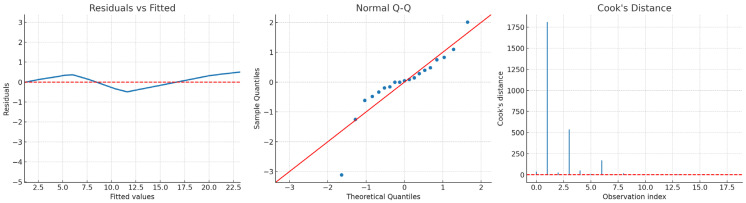
Diagnostic plots for the model (Equation (3)).

**Figure A7:**
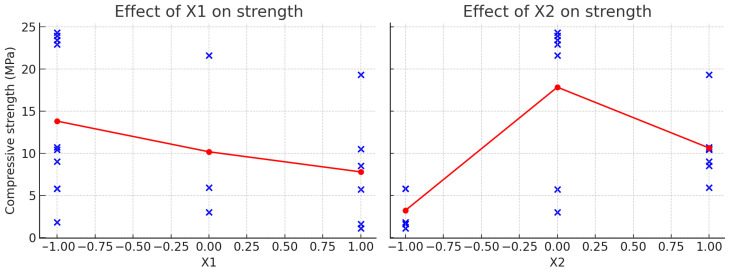
Univariate dependencies to the model (Equation (3)).

**Figure A8:**
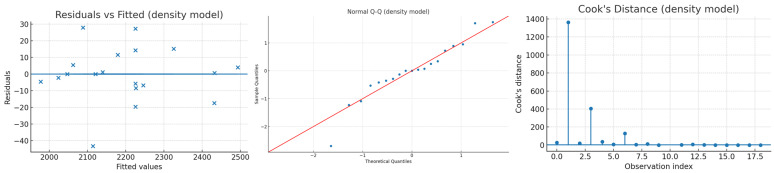
Diagnostic plots for the model (Equation (4)).

**Figure A9:**
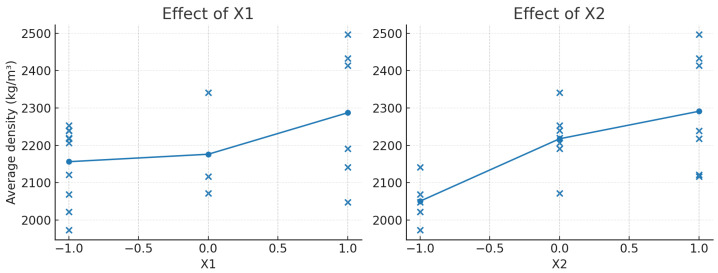
Univariate dependencies to the model (Equation 4).

**Figure A10:**
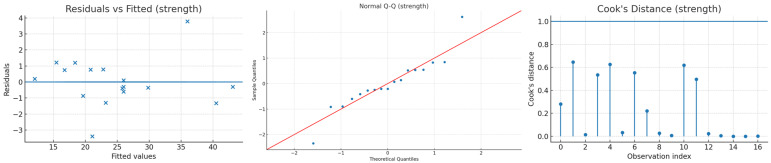
Diagnostic plots for the model (Equation (5)).

**Figure A11:**
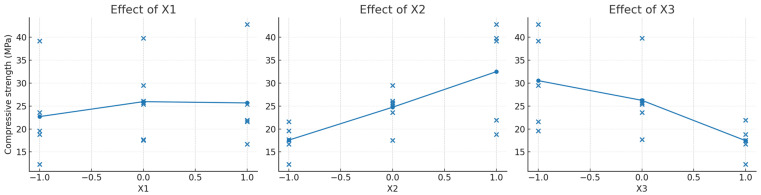
Univariate dependencies to the model (Equation 5).

**Figure A12:**
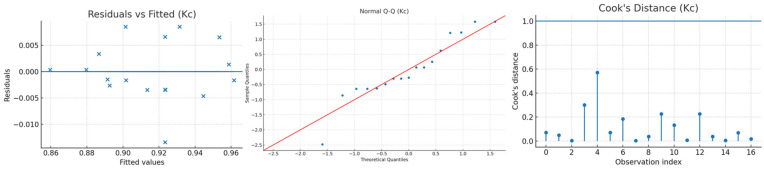
Diagnostic plots for the model (Equation (6)).

**Figure A13:**
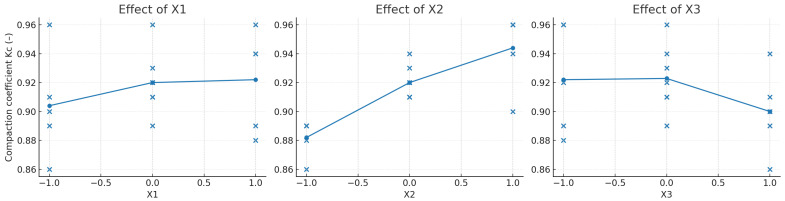
Univariate dependencies to the model (Equation (6)).

**Figure A14:**
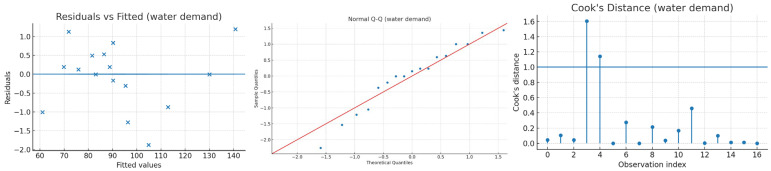
Diagnostic plots for the model (Equation (7)).

**Figure A15:**
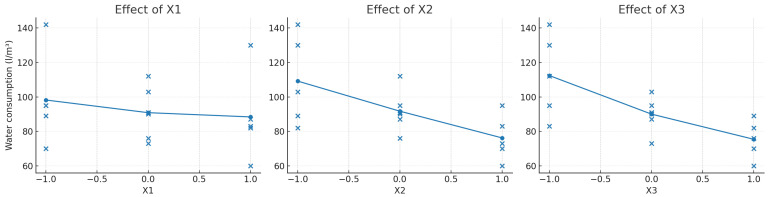
Univariate dependencies to the model (Equation (7)).

## Data Availability

The original contributions presented in this study are included in the article. Further inquiries can be directed to the corresponding author.

## Appendix B. Statistical Indicators of the Experimental–Statistical Model of the Gypsum Concrete Strength (Equation (1)) According to Plan B_4_ (Table 4)

**Table A1:** Full ANOVA table.

Source	Sum of Squares (SS)	df	F-Value	*p*-Value
X_1_	84.98	1	27.09	0.00022
X_2_	110.06	1	35.09	0.00007
X_3_	16.44	1	5.24	0.0410
X_4_	48.05	1	15.32	0.00206
X_1_^2^	42.43	1	13.53	0.00316
X_2_^2^	2.93	1	0.93	0.353
X_3_^2^	7.85	1	2.50	0.140
X_4_^2^	14.96	1	4.77	0.0496
X_1_·X_2_	11.27	1	3.59	0.0823
X_1_·X_3_	5.70	1	1.82	0.203
X_1_·X_4_	7.14	1	2.28	0.157
X_2_·X_3_	0.43	1	0.14	0.719
X_2_·X_4_	0.10	1	0.03	0.861
X_3_·X_4_	0.11	1	0.04	0.852
Residual	37.64	12	–	–
lack-of-fit	37.59	10	67.1	0.061
Pure error	0.045	2	–	–

Statistical indicators:

R^2^ = 0.935; Adjusted R^2^ = 0.859;

Lack-of-fit: F-value = 67.14, *p*-value = 0.061 (model adequate).

## Appendix C. Statistical Indicators of the Experimental–Statistical Model of the Average Concrete Density (Equation (2)) According to Plan B_4_ (Table 4)

**Table A2:** Full ANOVA table.

Source	Sum of Squares (SS)	df	F-Value	*p*-Value
X_1_	82.433	1	22.93	0.0003
X_2_	635.219	1	176.54	<0.0001
X_3_	14.682	1	4.08	0.063
X_4_	107.122	1	29.75	<0.0001
X1^2^	2.551	1	0.71	0.412
X2^2^	13.127	1	3.65	0.077
X3^2^	1.435	1	0.40	0.535
X4^2^	48.319	1	13.43	0.002
X_1_·X_2_	92.225	1	25.65	<0.0001
X_1_·X_3_	2.376	1	0.66	0.428
X_1_·X_4_	2.058	1	0.57	0.462
X_2_·X_3_	1.904	1	0.53	0.476
X_2_·X_4_	7.569	1	2.10	0.166
X_3_·X_4_	22.823	1	6.34	0.021
Residual Error	43.152	12	–	–
Lack-of-fit	34.012	8	27.7	0.0353
Pure Error	7.215	4	–	–

Statistical indicators:

R^2^ = 0.958; Adjusted R^2^ = 0.899;

Lack-of-fit: F-value = 27.74, *p*-value = 0.0353 (model adequate).

## Appendix D. Statistical Indicators of the Experimental–Statistical Model of the Concrete Strength (Equation (3)) According to Plan “Mixture-Technology-Property” (Table 5)

**Table A3:** Full ANOVA table.

Source	Sum of Squares (SS)	df	F-Value	*p*-Value
V_1_	2.44	1	0.23	0.0657
V_2_	38.94	1	3.67	0.0128
V_3_	13.26	1	1.25	0.0326
V_1_·V_2_	48.11	1	4.54	0.0100
V_1_·V_3_	315.24	1	29.73	0.0055
V_2_·V_3_	64.73	1	6.10	0.0689
X_1_	0.0	1.0	0.0	1.0
X_2_	0.0	1.0	0.0	1.0
X_1_^2^	217.18	1	20.48	0.0106
X_2_^2^	47.34	1	4.46	0.0102
X_1_·X_2_	0.0096	1	0.0009	0.0977
V_1_·X_1_	48.18	1	4.54	0.0100
V_2_·X_1_	49.97	1	4.71	0.0096
V_3_·X_1_	3.09	1	0.29	0.0618
V_1_·X_2_	74.47	1	7.02	0.0570
V_2_·X_2_	44.08	1	4.16	0.0111
V_3_·X_2_	21.65	1	2.04	0.0226
Residual	42.42	4	—	—
lack-of-fit	41.31	1	111.91	0.0018
Pure error	1.1075	3	—	—

Statistical indicators:

R^2^ = 0.967, Adjusted R^2^ = 0.850;

Lack-of-fit: F-value = 111.91, *p*-value = 0.0018 (model adequate).

## Appendix E. Statistical Indicators of the Experimental–Statistical Model of the Average Concrete Density (Equation (4)) According to Plan “Mixture-Technology-Property” (Table 5)

**Table A4:** Full ANOVA table.

Source	Sum of Squares (SS)	df	F-Value	*p*-Value
V_1_	707,685.7312	1.0	579.2658	0.0
V_2_	1,163,105.803	1.0	952.0433	0.0
V_3_	750,673.3224	1.0	614.4527	0.0
V_1_·V_2_	1123.5375	1.0	0.9197	0.3919
V_1_·V_3_	20,144.1141	1.0	16.4887	0.0153
V_2_·V_3_	4888.563	1.0	4.0015	0.1161
X_1_	0.0	1.0	0.0	1.0
X_2_	0.0	1.0	0.0	1.0
X_1_^2^	23,108.2937	1.0	18.915	0.0122
X_2_^2^	29,052.9009	1.0	23.7808	0.0082
X_1_·X_2_	9128.0402	1.0	7.4716	0.0523
V_1_·X_1_	47.1474	1.0	0.0386	0.8538
V_2_·X_1_	11,528.4865	1.0	9.4365	0.0372
V_3_·X_1_	5905.3496	1.0	4.8337	0.0928
V_1_·X_2_	1713.66	1.0	1.4027	0.3018
V_2_·X_2_	147.781	1.0	0.121	0.7455
V_3_·X_2_	999.0115	1.0	0.8177	0.417
Residual	4886.7771	4.0	–	–
lack-of-fit	3582.0271	1	8.2361	0.0641
Pure error	1304.75	3	–	–

Statistical indicators:

R^2^ = 0.987; Adjusted R^2^ = 0.941;

Lack-of-fit: F-value = 8.23 4, *p*-value = 0.064 (the model can be considered adequate).

## Appendix F. Statistical Indicators of the Experimental–Statistical Model of the Concrete Strength (Equation (5)) According to Plan B_3_ (Table 7)

**Table A5:** Full ANOVA table.

Source	Sum of Squares (SS)	df	F-Value	*p*-Value
X_1_	22.201	1.0	4.3644	0.0751
X_2_	556.516	1.0	109.4026	0.06
X_3_	429.025	1.0	84.3398	0.071
X_1_^2^	7.6982	1.0	1.5133	0.2584
X_2_^2^	17.4892	1.0	3.4381	0.1061
X_3_^2^	19.4604	1.0	3.8256	0.0914
X_1_·X_2_	0.0112	1.0	0.0022	0.9638
X_1_·X_3_	0.4512	1.0	0.0887	0.7745
X_2_·X_3_	105.8512	1.0	20.8087	0.0026
Residual	35.6081	7.0	—	—
lack-of-fit	35.36	5.0	57.342	0.0572
Pure error	0.1233	2.0	—	—

Statistical indicators:

R^2^ = 0.9701; Adjusted R^2^ = 0.9316;

Lack-of-fit: F-value = 57.34, *p*-value = 0.0572 (model adequate).

## Appendix G. Statistical Indicators of the Experimental–Statistical Model of the Compaction Coefficient (Equation (6)) According to Plan B_3_ (Table 7)

**Table A6:** Full ANOVA table.

Source	Sum of Squares (SS)	df	F-Value	*p*-Value
X_1_	0.001812	1	8.1991	0.0243
X_2_	0.002112	1	9.5546	0.0180
X_3_	0.000050	1	0.2258	0.6482
X_1_^2^	0.000000	1	0.0001	0.9910
X_2_^2^	0.001012	1	4.5743	0.0709
X_3_^2^	0.000050	1	0.2258	0.6482
X_1_·X_2_	0.000800	1	3.6197	0.1008
X_1_·X_3_	0.000012	1	0.0549	0.8230
X_2_·X_3_	0.000050	1	0.2258	0.6482
Residual	0.001553	7	—	—
lack-of-fit	0.001181	5	0.5921	0.7248
Pure error	0.001979	7	—	—

Statistical indicators:

R^2^ = 0.9646; Adjusted R^2^ = 0.9191;

Lack-of-fit: F-value = 0.592, *p*-value = 0.725 (model adequate).

## Appendix H. Statistical Indicators of the Experimental–Statistical Model of the Water Consumption (Equation (7)) According to Plan B_3_ (Table 7)

**Table A7:** Full ANOVA table.

Source	Sum of Squares (SS)	df	F-Value	*p*-Value
X_1_	826.281	1	198.876	0.000003
X_2_	1118.366	1	269.226	0.000001
X_3_	1089.366	1	262.037	0.000001
X_1_^2^	14.758	1	3.549	0.094676
X_2_^2^	52.758	1	12.678	0.009282
X_3_^2^	10.758	1	2.585	0.145734
X_1_·X_2_	19.778	1	4.756	0.065364
X_1_·X_3_	16.278	1	3.911	0.085158
X_2_·X_3_	21.278	1	5.122	0.058237
Residual	20.788	5	—	—
lack-of-fit	19.95	5	6.629	0.1358
Pure error	1.202	2	—	—

Statistical indicators:

R^2^ = 0.9983; Adjusted R^2^ = 0.9961;

Lack-of-fit: F-value = 6.63, *p*-value = 0.136 (model adequate).
